# Progress of Diphtheria in Chicago

**Published:** 1889-03

**Authors:** Charles Warrington Earle

**Affiliations:** Chicago, Ill.


### PROGRESS OF DIPHTHERIA IN CHICAGO.

BY CHARLES WARRINGTON EARLE, M. D., CHICAGO.

We have no record of the health of the early settlers of our city Cholera was brought to us in 1832 from Quebec, and during the following year, 1833, the town authorities began to pay attention to the Chicago river, by the declaration that it was unlawful to throw or put into the said river any carcass of dead animals under a penalty of $3.00. In 1834 the first Board of Health was established, but its existence was short and its functions exceedingly limited. From this time till 1859 (25 years), the word "diphtheria" does not occur in any reports to which I have access. May 9, 1837, Dr. Daniel Brainard was appointed Health Officer, and in 1841 an attempt was made to collect and have recorded the disease of which every person died. No record, however, can at this day be found.

In 1851 records of mortality were commenced and deaths from croup tabulated. It is of course difficult at this time to say that some of these cases were not diphtheritic.

From 1859 to the present time diphtheria has always been with us, the death rate ranging from 78, the lowest in 1867 to 1002, the highest in 1887.

Since 1876 there has been a marked increase in the number of deaths from diphtheria. Of course our population has increased very greatly; but the death rate from this disease is beyond what it should be. The death rate from all causes has diminished during the past five years, yet increased from diphtheria.

For instance, in 1868 the mortality was 23 in 1,000 population; 1869 the mortality was 23 in 1,000 population; 1870 the mortality was 24 in 1,000 population; 1871 the mortality was 20 in 1,000 population; 1872 the mortality was 27, average 23.

While in 1883 the mortality was 19; in 1884 the mortality was 19; in 1885 the mortality was 18; in 1886 the mortality was 19; n 1887 the mortality was 20; average 19.

In 1868, population 252,054, there were 87 deaths from diphtheria, or one in 2,897 population.

In 1873, population 380,000, 92 deaths from diphtheria, or one in 4,130 population.

In 1876, population 420,000, 464 deaths from diphtheria, or one in 905 population.

For five years, or till 1880, there was a steady increase in the number of deaths.

In 1880, population 503,298, deaths from diphtheria 930, or one in 541 population. This was the greatest mortality reached.

In 1884, population 630,000, 649 deaths from diphtheria, or 1 in 970.

In 1887, population 760,000, 1,002 deaths from diphtheria, or one in 758.

In 1888, population 810,000 to 850,000, with suburban towns included, 858 deaths from diphtheria, or 1 in 946 to 990, the lowest mortality in twelve years.

The greatest mortality has been until recently in the poorer wards, where people lived below grade and were crowded into houses. For instance, in 1880, the mortality in the 14th and 15th wards was 291 out of a total death rate from diphtheria, 930, $\frac{35}{109}$ of all the deaths. The mortality in the Stock Yard ward has always been high. This has been brought about because of the large population in these wards and bad hygienic surroundings. These conditions always give a large mortality when infection has once taken place. The sanitary condition of these wards has been greatly improved, particularly in raising the grade, increasing the sewerage and personal inspection on the part of the health officers.

It has now come to be not an unmixed blessing to live in a good ward, for the truly good have been allowed to do as they please, and the poor are inspected and their habitations made clean. As a result, in the year when the mortality has been the lowest throughout the city (1888), one death from diphtheria in about 950, we have had in the 10th, 11th and 12th wards 132 deaths, and in the 15th, 16th and 17th wards (the old 14th), 126 deaths.

What we need, then, at this time is greater precaution in the way of isolation and sanitary watchfulness; and I am not sure but the greatest good which this Society could do in regard to this disease would be to commence an agitation which would give to our Health Department full and complete control of it. Personally, I am of the opinion that the time has come for the city to take charge of every house infected with this terrible scourge.

